# Is it them or is it us? Unravelling ethnic disparities in undergraduate clinical performance

**DOI:** 10.1186/s12916-017-0959-5

**Published:** 2017-10-25

**Authors:** Karen M. Stegers-Jager

**Affiliations:** Institute of Medical Education Research Rotterdam, Erasmus MC, University Medical Centre Rotterdam, Room AE-241, PO Box 2040, 3000 CA, Rotterdam, Netherlands

**Keywords:** Medical education, Assessment, Differential attainment, Ethnicity, Stereotypes, Communication styles

## Abstract

Given our increasingly diverse societies, there is an urgent need for research into the causes of persistent ethnic disparities in undergraduate clinical performance. It is argued that causes for underperformance can be identified from two perspectives, namely that of the students (‘them’) and that of the academic environment (‘us’). Taking the ‘us’ perspective, Yeates et al. conducted a detailed experimental study aimed at understanding the processes underlying judgment and decision-making in clinical assessments. Contrary to their expectations, their study indicates that, despite the presence of active stereotypes, examiner bias does not explain ethnic minority students’ underperformance. Naturally, future studies are required to confirm their findings. It is suggested that these studies should take into account various rater and situational factors (e.g. rater experience, increased cognitive load) that may influence examiners’ reliance on stereotypes. However, future work should also focus on other potential impeding factors from both perspectives, including differences in communication styles. Knowing what leads to the ethnic disparities in performance is a prerequisite for designing interventions aimed at ensuring a level playing field for a diverse student population.

Please see related article: https://bmcmedicine.biomedcentral.com/articles/10.1186/s12916-017-0943-0

## Background

The increase in ethnic and cultural diversity of many western societies in recent decades has resulted in growing numbers of minority group patients and in increasingly diverse student populations in medical schools. As a result of these developments, the field of medical education faces several challenges. The main challenge is that of increasing diversity in the healthcare workforce in order to improve healthcare access and quality for minority patients [1].

Increasing diversity not only serves the cause of social justice, but also makes good business sense [2] since diversity and inclusion lead to excellence [3]. However, the pursuit of increasing diversity is hampered by the persistent ethnic disparities in medical school performance. Numerous studies, including our own [4], have shown that medical students from ethnic minorities underperform compared with those from the ethnic majority, particularly on clinical (rater-based) assessments, even when controlled for prior attainment [4, 5]. As clinical grades are known to play a key role in selection for residency training, lower clinical grades for ethnic minority students may contribute to their underrepresentation in residency training and in the medical workforce. Unfortunately, thus far, little is known about why this underperformance occurs and how to address it [5].

As underperformance can be seen as the result of a mismatch between the student and the academic environment, explanations can be identified from both perspectives, i.e. is it them or is it us? Put differently, do ethnic minority students actually perform worse or do their assessors think they do so? Knowing what leads to ethnic disparities in performance is a prerequisite for designing interventions aimed at ensuring fair clinical grades for ethnic minority students.

## Recent evidence

Yeates et al. [6] assessed whether examiners are biased against ethnic minority students, i.e. from the ‘us’ perspective, thus providing information about the potential of examiner bias in an experimental study. Participants, namely UK Objective Structured Clinical Examination (OSCE) examiners, were assigned to watch scripted videotaped OSCE performances by Caucasian and Asian students that were either consistent or inconsistent with a previously described stereotype of Asian students’ performance. Surprisingly, their hypotheses regarding the presence of bias were not confirmed, despite the fact that these originated from a whole body of existing literature from the social psychology domain. The Asian students did not receive lower scores than Caucasian students, and the feedback provided to the Asian students was also not comparatively negative nor more focused on communication skills than that given to Caucasian students.

Nevertheless, using a lexical decision task, Yeates et al. [6] did find evidence of the presence of Asian stereotypes in examiners’ mind. Contrary to their expectations, this occurred regardless of whether examiners observed stereotype-consistent or -inconsistent performances. Furthermore, in absence of a control group, we cannot tell whether the stereotypes were activated by the task at hand or were already present beforehand. In any case, it is remarkable that the presence of active stereotypes did not influence examiners’ scores, their feedback or their memories of performances.

## Explanations, implications and further research

Why did Yeates et al. [6] not find an effect of stereotype bias in their study? As suggested by the authors themselves, it might be that the raters were able to resist stereotyping. A theory that can help elucidate how students’ ethnic minority status affects raters’ information processing during assessment is the dual-process theory [7] – one of the most fundamental models on judgment and decision-making in social and cognitive psychology [8]. Dual-process theory distinguishes two information processing modes, namely a fast, automatic mode (‘System 1’) and a deliberate, conscious mode (‘System 2’). System 1 is considered to use mental shortcuts (heuristics), which are prone to cognitive biases. Such biases, including the activation of stereotypes and prejudiced attitudes, might result in lower scores for ethnic minority students [7]. System 2 executes complex cognitive operations and monitors the outcome or responses of System 1. Whether System 2 intervenes in the case of undesirable System 1 impulses depends on situational factors and rater characteristics [7].

Rater factors that might increase reliance on System 1 processes are their prejudiced attitudes, their (perceived) level of experience and their ‘need for cognitive closure’ – a personality characteristic related to being decisive and closed-minded [7]. Meanwhile, (perceived) similarity to the student and concerns about prejudice might decrease a rater’s risk of bias [7]. Building in accountability, structuring the assessment, and the presence of diversity policies and training are likely to stimulate System 2 processes, whereas high cognitive demands and pressure hamper the correction of initial impressions [7].

Thus, it could well be that the sense of professionalism or experience of working with ethnic minority students helped the participants in Yeates’ study to resist reliance on System 1 processes, i.e. stereotyping. On the other hand, one should be aware that experience and a sense of objectivity (‘I think it, therefore it is true’) caused by the ‘position of power’, which is characteristic for medical doctors, could also lead to a stronger reliance on System 1 processes [9]. Future research into unravelling the judgment process of raters should explore the influence of experience and the other mentioned rater and situational factors. For example, what would be the effect of increased pressure and higher cognitive demands (e.g. longer series of student performances) as occurs in actual practice of OSCE examinations? What would happen when ethnic minority students are assessed by ethnic minority raters? Would their scores be higher, similar or even lower due to ‘self-group distancing’ by raters who are individually successful members of their group? [10] Would there be a mitigating effect of the scoring method used?

Another reason why the consistently reported underperformance of ethnic minority students was not confirmed in the study by Yeates et al. [6] may be related to differences in communication styles. Yeates et al. used scripted videos with nearly identical Caucasian and Asian versions. However, the question is whether the communication skills of Asian students in real OSCEs are poorer (as suggested by the described stereotype [11]) or just different. In other words, is there just one correct way of interacting with patients? Do all patients favour the same communication styles? A study by Wass et al. [12] on examiner bias in a final year OSCE suggested that opinions on what was considered good interaction differed between Caucasian examiners and ethnic minority simulated patients. Other studies showed that levels of assertiveness and reticence [13] and patient-centeredness [14] were related to clinical grades. However, even within Western Europe, there are differences in doctor-patient communication preferences [15]. In preparing our future doctors for an increasingly diverse patient population we may have to reconsider the existence of one golden standard for doctor-patient communication.

## Conclusions

There is an urgent need for research into the causes of ethnic disparities in undergraduate clinical performance. Yeates et al. [6] responded to the call for more detailed experimental studies to understand the processes underlying judgment and decision-making in clinical assessments. Although their study indicates that examiner bias does not explain ethnic minority students’ underperformance, future studies – preferably taking into account various rater and situational factors (e.g. experience, longer series of performances) – are required to confirm their findings. Additionally, other potential impeding factors from both perspectives, including differences in communication styles, should be studied and accompanied by possible interventions in order to ensure a level playing field for a diverse student population.
